# Hybrid modeling frameworks of tumor development and treatment

**DOI:** 10.1002/wsbm.1461

**Published:** 2019-07-17

**Authors:** Ibrahim M. Chamseddine, Katarzyna A. Rejniak

**Affiliations:** ^1^ Department of Integrated Mathematical Oncology H. Lee Moffitt Cancer Center and Research Institute Tampa Florida; ^2^ Department of Oncologic Sciences, Morsani College of Medicine University of South Florida Tampa Florida

**Keywords:** mathematical modeling, mathematical oncology

## Abstract

Tumors are complex multicellular heterogeneous systems comprised of components that interact with and modify one another. Tumor development depends on multiple factors: intrinsic, such as genetic mutations, altered signaling pathways, or variable receptor expression; and extrinsic, such as differences in nutrient supply, crosstalk with stromal or immune cells, or variable composition of the surrounding extracellular matrix. Tumors are also characterized by high cellular heterogeneity and dynamically changing tumor microenvironments. The complexity increases when this multiscale, multicomponent system is perturbed by anticancer treatments. Modeling such complex systems and predicting how tumors will respond to therapies require mathematical models that can handle various types of information and combine diverse theoretical methods on multiple temporal and spatial scales, that is, *hybrid models*. In this update, we discuss the progress that has been achieved during the last 10 years in the area of the hybrid modeling of tumors. The classical definition of hybrid models refers to the coupling of discrete descriptions of cells with continuous descriptions of microenvironmental factors. To reflect on the direction that the modeling field has taken, we propose extending the definition of hybrid models to include of coupling two or more different mathematical frameworks. Thus, in addition to discussing recent advances in discrete/continuous modeling, we also discuss how these two mathematical descriptions can be coupled with theoretical frameworks of optimal control, optimization, fluid dynamics, game theory, and machine learning. All these methods will be illustrated with applications to tumor development and various anticancer treatments.

This article is characterized under:

Analytical and Computational Methods > Computational Methods
Translational, Genomic, and Systems Medicine > Therapeutic Methods
Models of Systems Properties and Processes > Organ, Tissue, and Physiological Models

## Graphical abstract

Hybrid modeling framework of cancer combines three classes of mathematical models: data‐driven, physics‐based, and optimization for development of a clinically relevant and quantitative decision‐making system for personalized medicine.

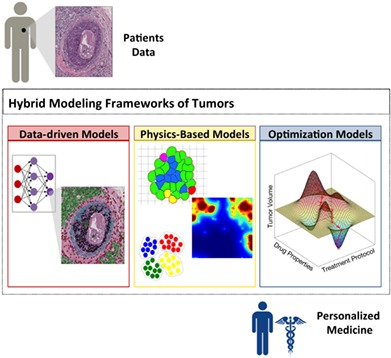

## 1 INTRODUCTION

It is now widely accepted that tumors are complex, heterogeneous, and multiscale. Tumor development spans several spatial and temporal scales. The genetic changes and modifications in intracellular protein expression act on an atomic scale and can be accomplished in milliseconds. On the molecular scale, signaling events and cellular processes can take seconds. The cell cycle progression and cell interactions with other cells and with the microenvironment comprise the microscopic scale and can be counted in minutes. The macroscopic tissue‐level effects are noticeable on the scale of hours. Finally, changes on the whole‐body level and patients' observable responses to treatments can take months or years and can be described on the population scale. Tumor heterogeneity refers to the diversity of cellular and microenvironmental components of the tumor, to differences between individual tumor cells, and to spatial variability in tumor composition, including microenvironmental gradients and tissue architecture. These various scales and heterogeneity phenomena require the use of different mathematical techniques for an adequate description of and proper solution to each component, as well as for effective coupling between components. Hybrid models are capable of addressing this complexity of tumors.

### 1.1 Classical hybrid models

**Figure 1:**
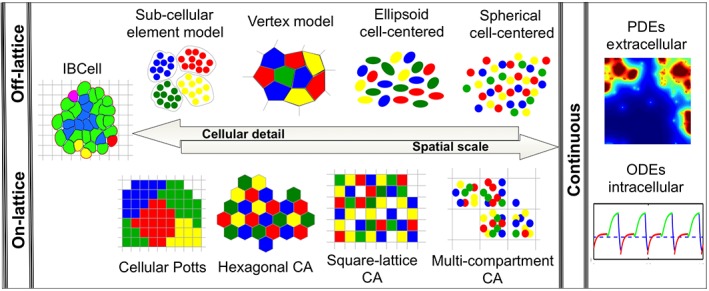
Schematic representation of the components of the classical hybrid models. The discrete components (on‐lattice and off‐lattice) show reciprocal relation between the number of cells handled by each modeling technique and the level of included cellular details. In each class, the model complexity rises from cells represented by single points to fully deformable bodies. The continuous components describe either time‐dependent intracellular molecular kinetics (ODEs) or time‐ and space‐dependent extracellular molecular dynamics (PDEs)

In our previous review (Rejniak & Anderson, 2011), we described the classical hybrid models that combine discrete equations to describe individual cells (tumor, stromal, immune) and continuous equations for microenvironmental factors (diffusible nutrients or extracellular matrix [ECM]‐bound growth factors) or intracellular components (proteins and enzymes). In these classical models, the extracellular factors are often traced in time and space in the whole computational domain using partial differential equations (PDEs) and are solved on regular or unstructured grids. The intracellular changes are followed separately for each cell using time‐dependent ordinary differential equations (ODEs). The individual cells (agents) are well‐suited for modeling even small differences between separate cells. Thus, it is relatively easy to incorporate tumor heterogeneity into these models. How the exchange of information between these two mathematical frameworks is implemented depends on whether the discrete cells are defined on‐ or off‐lattice and whether the continuous equations are solved on a square, hexagonal, or triangular grid. Several distinct agent‐based models have been developed so far. The graphical summary of these classical hybrid models is shown in Figure 1. For more details, we refer the reader to our previous review (Rejniak & Anderson, 2011).

### 1.2 Hybrid modeling frameworks

**Figure 2:**
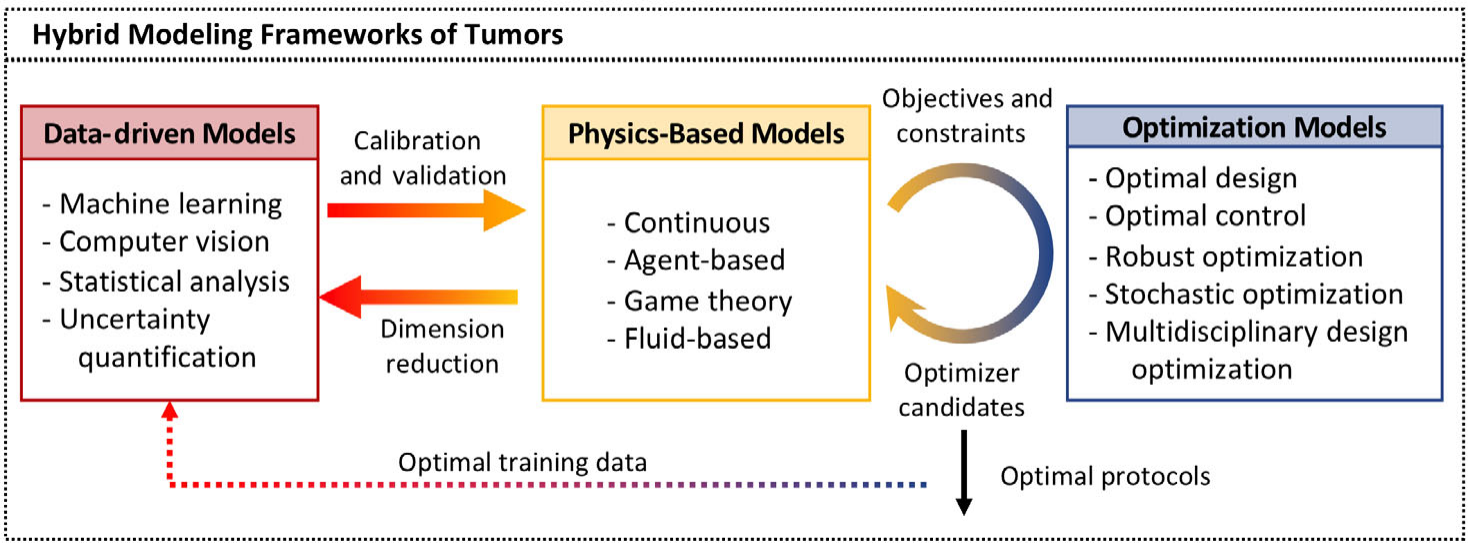
Schematics of hybrid modeling frameworks of tumors. Three classes of models are used for cancer problems: data‐driven, physics‐based, and optimization. Combination of models from any two classes is considered a hybrid modeling framework. The solid arrows represent interactions between different frameworks that are currently modeled. The dotted arrow represents a promising future direction. The classical hybrid models belong to the class of physics‐based models

In the past decade, the field of tumor modeling reached into other mathematical areas and combined continuous or discrete models with concepts from fluid dynamics, game theory, machine learning, or optimization to provide new predictive models of tumor development and response to treatments. Therefore, to reflect on the direction in which the tumor modeling community has moved, we propose here to extend the definition of hybrid models to include the coupling of two or more different mathematical frameworks. The concept of *hybrid modeling frameworks* is summarized graphically in Figure 2.

The classical hybrid discrete/continuous models belong to the physics‐based category of mathematical models, in which the behavior of modeled entities is based on physical laws and principles. Other models in this group are fluid‐based models that use fluid flow principles (conservation of mass and momentum) to model either the blood flow in the vasculature, the interstitial fluid flow in the extracellular matrix, or the cytoplasm inside the cells. Finally, the game theory models are used to test strategic interactions between model components. In particular, this class of models was used to address the emergence of drug resistance that allowed for developing a concept of adaptive therapy.

In recent years, other types of mathematical models were introduced to the field of mathematical oncology. With the increased number of available pre‐clinical and clinical data, there is a need to develop suitable methods for their analysis. The data‐driven models use statistical correlations and quantitative algorithms to accurately identify, classify, and find patterns in the underlying data. Such models can be combined with physics‐based models to provide data for calibration and validation. In this context, the data‐driven models (machine learning or computer vision) are most often applied to medical imaging data, such as immunohistological imaging, fluorescent imaging, magnetic resonance imaging (MRI), computed tomography (CT), or positron‐emission tomography (PET). On the other hand, the uncertainty quantification models provide a strong tool for representing, propagating, and inferring uncertainties both in the experimental or clinical data and in model parameters. These models have the potential to enhance the robustness, confidence in and predictability of physical models. Moreover, the integration of data‐driven models with physics‐based models can be expanded to applications where there are large clinical and experimental data sets. The physics‐based models can be used to streamline the analysis and interpretation of clinical data by applying constraints in order to reduce data dimensionality before data‐driven models are developed.

The physics‐based models are often computationally expensive, which may prohibit the exploration of the broad model parameter space and the determination of the most effective treatment schedules. The optimization models integrated with the physics‐based models can provide a way to systematically search for optimal treatment protocols using a minimal number of iterations. Among optimization methods are optimal design, which is a selection process from a set of available alternatives based on the objective functions and constraints; optimal control which aims to find the laws for a time‐dependent dynamic system so that the performance of the system is optimal with respect to some criterion; multi‐objective optimization models that provide a solution to quantifying tradeoffs between competing objective functions, such as maximizing tumor death and minimizing drug resistance; robust and stochastic optimization methods that consider uncertainties in the data or the model while generating robust optimal protocols applicable to wide cohorts of mice or patients.

**Figure 3:**
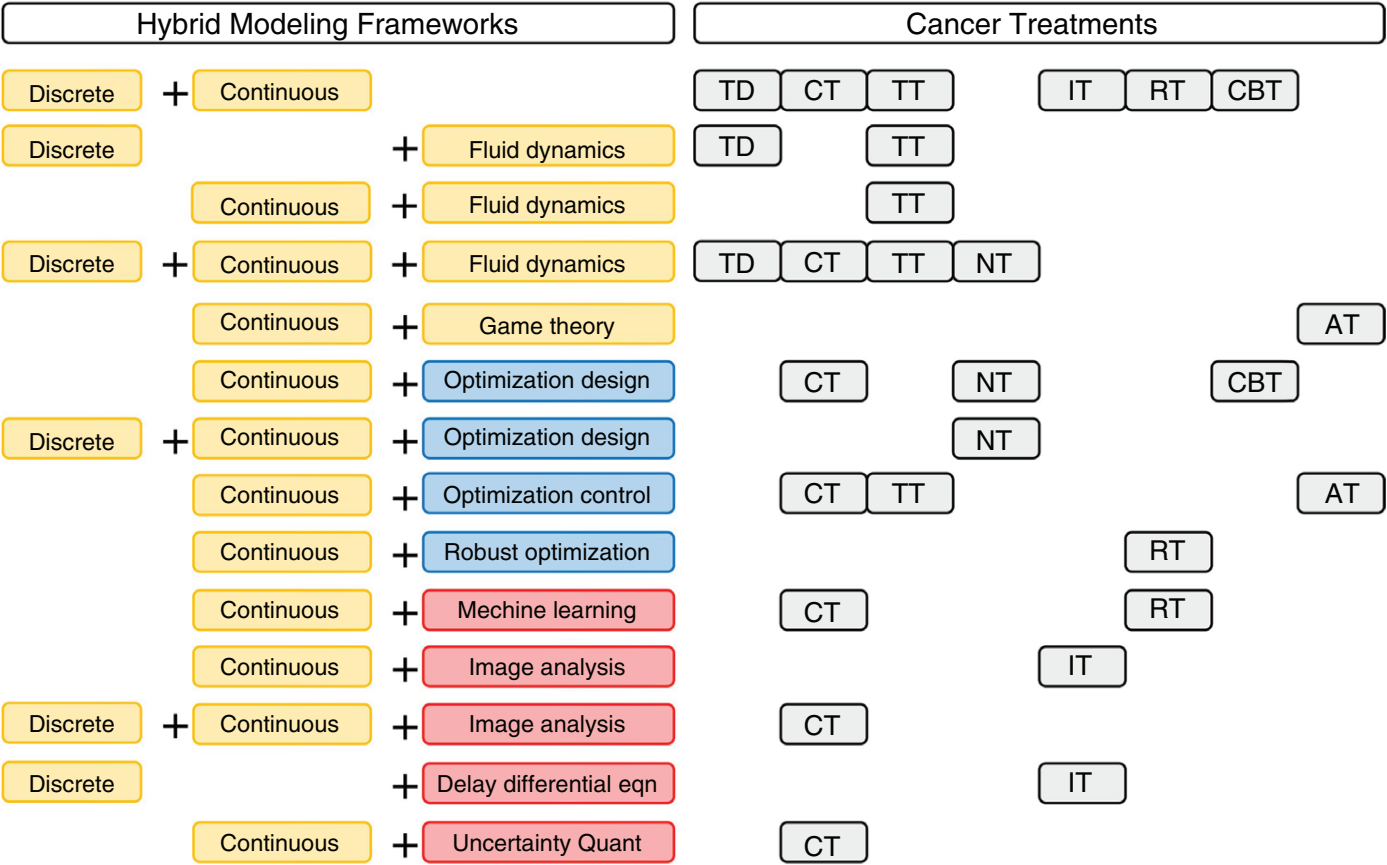
Summary of the discussed hybrid modeling frameworks. Letter codes denote a section in which the given models were cited: AT, adaptive; CBT, combination; CT, chemotherapy; HT, hormone; IT, immunotherapy; NT, nanotherapy; RT, radiation; TT, targeted therapy and TD, tumor development

In this review, we presented the recent advances in discrete/continuous tumor modeling and the coupling of either discrete or continuous models with other mathematical theories to form hybrid modeling frameworks. These mathematical models are discussed in the context of their application to anticancer treatments. Figure 3 provides a guide to the hybrid modeling frameworks discussed in this review.

## 2 MODELING TUMOR DEVELOPMENT

Solid tumor growth is initiated within a normal tissue structure, such as epithelial ducts (carcinomas), skin epithelial multilayers (melanomas), the supportive tissues of the body (sarcomas), or the brain tissues (gliomas). Tumor spread to distant sites (metastasis) is preceded by the development of new tumor vasculature (angiogenesis), tumor invasion through the surrounding stroma, interactions with stormal and immune cells and exposure to variable and dynamically changing levels of metabolites and cytokines. The hybrid frameworks that discuss the role of these factors in tumor development and progression are presented below.

**Figure 4:**
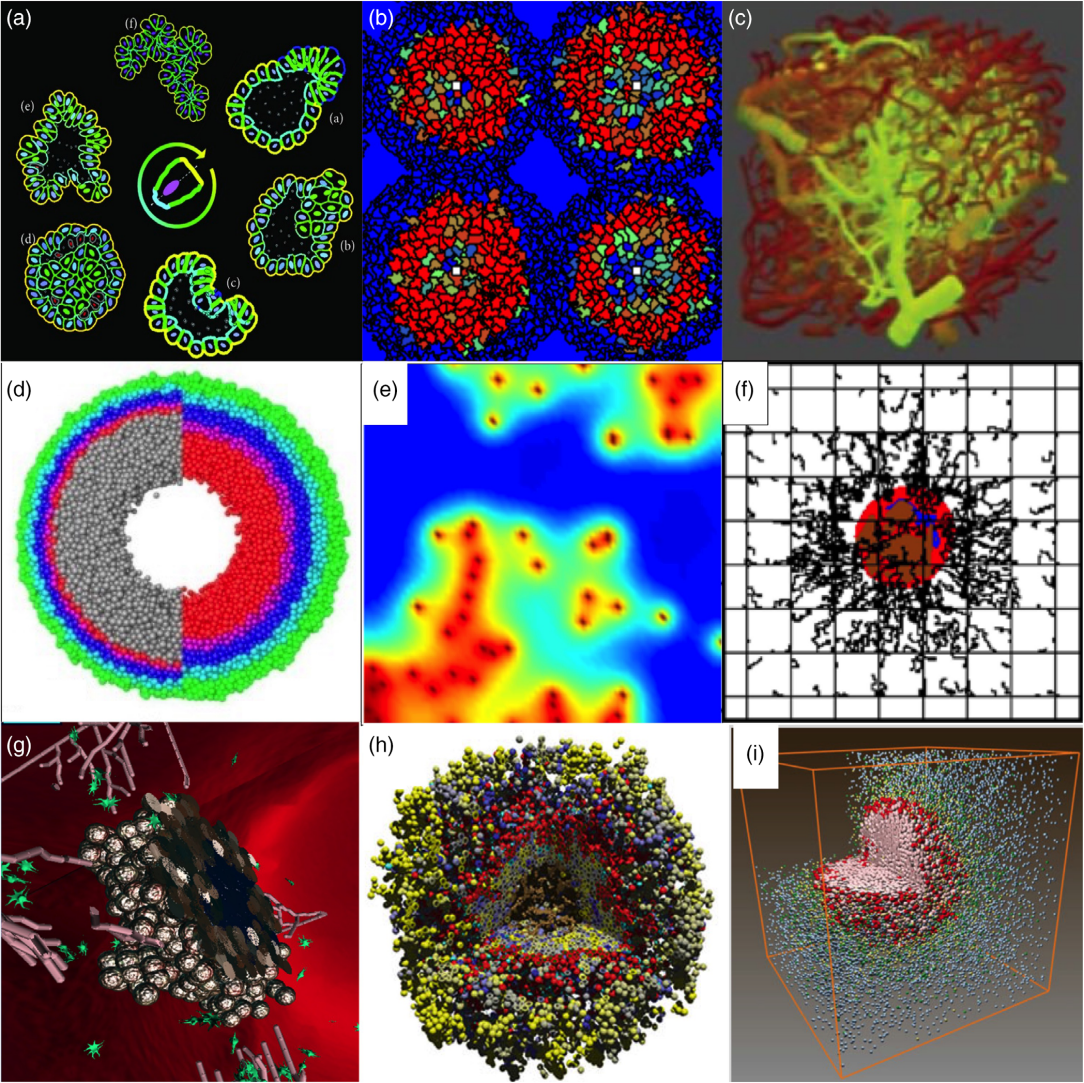
Snapshots from simulations of various hybrid frameworks of tumor development and treatment. (a) Fluid‐based model of individual deformable cells (Reprinted with permission from Rejniak (2012). Copyright 2012 Hindawi Publishing Corporation); (b) Potts model combined with PDEs (Reprinted with permission from Szabo and Merks (2017). Copyright 2017 Public Library of Science); (c) image‐based vascular network model with flow and diffusion (Reprinted with permission from Boujelben et al. (2016). Copyright 2016 Royal Society); (d) particle‐spring model for radiation (Reprinted with permission from Kempf, Bleicher, and Meyer‐Hermann (2015). Copyright 2015 Public Library of Science); (e) CA model combined with PDEs (Reprinted with permission from Scott, Fletcher, Anderson, and Maini (2016). Copyright 2016 Public Library of Science); (f) discrete vasculature model combined with PDEs (Reprinted with permission from Chamseddine, Frieboes and Kokkolaras (2018). Copyright 2018 Springer Nature); (g) agent‐based model combined with PDEs (Reprinted with permission from Bloch and Harel (2016). Copyright 2016 Springer Nature); (h) particle‐spring model combined with PDEs (Reprinted with permission from Ghaffarizadeh, Heiland, Friedman, Mumenthaler, and Macklin (2018). Copyright 2018 Public Library of Science); (i) CA model combined with PDEs (Reprinted with permission from Gong et al. (2017). Copyright 2017 Royal Society)

The initial tumor growth is confined to the structure of the host tissue. For example, tumors developing in the breast ducts first fill the inner lumen and form ductal carcinomas in situ (DCIS). This early tumor stage was modeled by Macklin, Edgerton, Thompson, and Cristini (2012) using a classical off‐lattice discrete‐continuous hybrid approach. The authors reproduced the longitudinal cross section through the duct and modeled DCIS growth from its initiation to the formation of central microcalcifications. The authors also developed an algorithm for model calibration using patient‐specific histopathology data. The 3D version of this model is described in Ghaffarizadeh et al. (2018). A different hybrid framework was used by Rejniak et al. (2012) to model a circular cross section though a mammary acinus, with a 3D in vitro culture mimicking the breast terminal bud. This model combined discrete off‐lattice deformable cells with fluid dynamics equations for cytoplasm and an external medium that were solved on a regular grid (the *IBCell* model). The authors simulated normal epithelial ducts, as well as fully or partially filled ducts with multiple luminal spaces that resemble either the solid or the cribriform patterns of DCIS. In addition, the analysis method (the morphochart) was developed to generate predictions about the up‐ or down‐regulation of cellular traits based on the observed experimental morphologies. The same model was used in Rejniak (2012) to predict alterations in cell–cell signaling that led to the development of invasive clones within the simulated duct (Figure 4a). These microinvasions constitute the first step in the lethal metastatic cascade.

The availability of nutrients supplied from the pre‐existing vasculature limits the growth of avascular tumors. This barrier can be overcome by the sprouting of new vessels (angiogenesis) to ensure adequate oxygen and nutrient delivery. The angiogenesis process was simulated using the classical discrete‐continuous model by Stéphanou et al. (2017). In this model, both tumor cells and tumor vasculature were represented by lattice‐based cellular automaton (CA), while continuous equations were used to describe the kinetics of growth factors, oxygen, vascular endothelial growth factor (VEGF), and the proteases produced by tumor and endothelial cells (EC). The model, calibrated to data from the dorsal window mouse experiments, was used to simulate the tumor‐induced changes in angiogenesis. The obtained results suggested that vascular alternations can favor cell dormancy. This model was also used to develop a concept of a patient‐specific virtual tumor (Caraguel, Lesart, Estève, van der Sanden, & Stéphanou, 2016). A hybrid model combining the fluid dynamics of blood flow through a network of discrete vessel segments with a continuous description of the oxygen and VEGF fields was used by Pries and Secomb (2014) and Secomb, Alberding, Hsu, Dewhirst, and Pries (2013). The initial structure of the vascular network was calibrated to a rat vasculature acquired from intravital microscopy images. Model simulations showed that a sufficient nutrient coverage can be achieved when over‐abundant vessel generation is accompanied by vessel refinement caused by structural adaptation and pruning. A different hybrid model combining a continuous description of a growing tumor with a discrete model of tumor vasculature based on a triangulated grid was used by Rieger, Fredrich, and Welter (2016) and Welter, Fredrich, Rinneberg, and Rieger (2016). This model also included equations for the vascular transport of hematocrit and oxygen and continuous equations for the interstitial diffusion of oxygen. The simulations tested distributions of oxygen and hemoglobin in tumor vasculature versus normal vasculature. They showed that concentrations below normal can only be achieved by reducing the tumor vessel radii during growth, which mimics vessel compression caused by intratumoral solid stress due to tumor growth.

The formation of new vasculature induced by the tumor allows for further tumor expansion and the emergence of invasive tumor cells that lead to the development of distant tumor metastases. Szabo and Merks (2017) used a hybrid approach that couples the on‐lattice Cellular Potts model of individual deformable cells and the continuous description of the concentrations of oxygen, glucose and lactate. This model was used to study the effect of blood flow obstruction on tumor evolution, showing that instabilities in blood supply can lead to the extinction of the aggressive tumor phenotypes (Figure 4b). Thus, not fitness at the cell level but the competition for nutrients and space determines the success of each tumor cell. Caiazzo and Ramis‐Conde (2015) used a hybrid model with discrete off‐lattice tumor cells and diffusion–reaction equations describing the oxygen kinetics within a 3D brain tissue to investigate the formation of glioblastoma palisade pattern. The simulations showed that while the response to hypoxia can explain the emergence of densely packed cell colonies (a palisade), it is not sufficient to explain the local invasion. The additional assumption of heterogeneity in cell sensitivity to hypoxia allowed for the reproduction of the patterns seen in histology slices. The experimentally observed melanoma tumors and vasculatures were reproduced by Łos, Paszynski, Kłusek, and Dzwinel (2017) using fast computational methods to implement a hybrid model combining a continuous description of tumor growth, ECM density, and tumor angiogenic factor with discrete segments representing a tumor vascular network.

As long as tumor cells are confined within a primary tumor mass, they often can be surgically resected. However, the lethal threat is presented by the cells that are able to escape the tumor mass and invade the surrounding stroma. These individual cells or small‐cell colonies are hard to identify during surgery and, when left untreated, may lead to tumor recurrence or metastasis. Andasari, Roper, Swat, and Chaplain (2012) combined the *CompuCell3D* (Cellular Potts model) and the *BioNetsolver* (an intracellular network model) to study the dynamics of cancer growth and invasion driven by the dynamics of E‐cadherin and β‐catenin. The authors tested how changes in either the association rate of β‐catenin or its degradation lead to an epithelial‐to‐mesenchymal transition and detachment of tumor cells from a layer of epithelial cells or from a multicellular tumor spheroid. A model of deformable individual cells (based on the fluid–structure interaction immersed boundary method) was used by W. Lee, Lim, and Kim (2017) to simulate the invasion of glioma cells through the brain tissue. The authors demonstrated that the coordination of biochemical and mechanical signals within a glioma cell enabled the cell to pass through narrow intercellular spaces and infiltrate the surrounding tissue.

Once tumor cells begin to move through the stroma surrounding the tumor, they encounter a new environment and start interacting with stromal cells. Ghaffarizadeh et al. (2018) used the 3D off‐lattice *PhysiCell* model to simulate an immune attack on the tumor. In this model, the immune cell migrated towards the cytokines secreted by tumor cells; thus, tumor recovery from the immune attack depended on the strength of the homing effect and the immune cells' persistence in moving towards the cytokine gradient. A similar model was presented by Bloch and Harel (2016) to simulate tumor cell interactions with stromal fibroblasts and EC in response to levels of oxygen and VGEF. The model reproduced tumor tissue architecture and tumor vasculature patterns comparable to those observed experimentally (Figure 4g). Moreover, the simulations identified a turning point (a combination of oxygen and VEGF levels) that separated tumor regression from its uncontrolled expansion. A more detailed investigation of tumor cell response to metabolite gradients was presented by Pérez‐Velázquez, Gevertz, Karolak, and Rejniak (2016) using a combination of the off‐lattice representation of individual tumor cells and vessels with reaction–diffusion PDEs that described metabolite kinetics. Model simulations showed that nonuniform tumor vasculature resulted in irregular gradients of oxygen and the drug and could lead to the emergence of specific spatial regions, hypoxic niches, or pharmacological sanctuaries. These tissue areas can shelter tumor cells and enable the development of resistant cell cohorts.

## 3 MODELING CHEMOTHERAPY

Chemotherapy is an anticancer treatment that employs cytotoxic or cytostatic drugs to reduce tumor burden by either killing tumor cells or suppressing their proliferation. Since this is a systemic treatment, it has the ability to affect both localized and metastatic tumors. However, tumor heterogeneity both cellular and metabolic can constitute a barrier to effective drug delivery. Moreover, chemotherapy drugs are not tumor cell‐specific and, therefore, one of the main concerns in the clinic is the systemic toxicity. The hybrid modeling frameworks that address these issues, predict the tumor response to chemotherapy and facilitate the design of more effective treatment protocols are presented below.

One of the main barriers to the efficient transport of chemotherapeutics is the structure of the tumor microenvironment. High interstitial pressure and abnormal vasculature creates a burden for the delivery of drugs to cancerous cells. These aspects of the tumor microenvironment were studied by Wu et al. (2013, 2014) using a classical hybrid model that couples an on‐lattice discrete angiogenesis model, tumor mass described as an incompressible fluid in a porous medium, and continuous equations for the transport of the drug and metabolites. The authors considered various physical conditions, such as vascular and interstitial hydraulic conductivities, osmotic pressure, and lymphatic drainage, in developing strategies that enhanced the anti‐tumor activity of chemotherapeutic drugs.

The obstacle specific to the delivery of chemotherapeutics to brain tumors is the blood–brain barrier (BBB). It remains uncertain whether the normalization of the BBB by anti‐angiogenic therapy enhances or limits the delivery of drugs to brain tumors. Boujelben et al. (2016) investigated how blood flow, vascular permeability, and diffusion within the tumor microenvironment affect drug delivery within the brain tumor. In this model, the vessels were treated as discrete lattice‐free elements with shared junctions, and the flow in each vessel was determined using a Poiseuille flow equation. The concentration of drugs in each vessel was updated based on its exchange with connected vessels and nearby tissue. The results predicted a nonlinear relation between the flow properties and drug concentration in the tissue (Figure 4c). Another complexity encountered by brain tumor therapies is the presence of highly migratory glioma cells. These cells cannot be fully removed during surgery, and thus are prone to the initiation of tumor recurrence or regrowth. Y. Kim et al. (2015) developed a hybrid multiscale model of migratory cells that combined lattice‐free cells with continuous equations of biochemical factors, such as oxygen, nutrients, and chemoattractants. This model was used to investigate the role of cell motility on tumor response to chemotherapy and to evaluate the efficacy of chemotherapy in reaching hidden migratory brain tumor cells.

Almost all chemotherapy treatments lead to the development of resistance. The role of heterogeneities in the tumor microenvironment on the emergence of resistant cell populations was modeled by Pérez‐Velázquez et al. (2016). The authors developed a hybrid model that couples off‐lattice tumor cells with reaction–diffusion equations of oxygen and drug gradients arising from irregularly placed vessels. The authors concluded that specific microenvironmental niches, such as drug sanctuaries characterized by low drug contents and normal oxygenation, allow tumor cells to develop drug tolerance without acquiring a lethal drug insult. A similar question was posited by Hamis, Nithiarasu, and Powathil (2018) using the on‐lattice hybrid model. The authors considered combinations of various intracellular, extracellular, and intercellular factors and simulated multiple chemotherapy strategies in order to demonstrate that optimal treatment protocols drastically depend on which drug‐resistant mechanisms are activated and that, furthermore, suboptimal chemotherapy administration may promote drug resistance.

One of the most critical aspects of chemotherapy treatment is its proper scheduling. This has a direct effect on tumor regression, relapse, and the evolution of resistant cells. To determine the optimal therapy protocols, one needs to consider the order of the drugs, their timing and frequency of administration, and the dosage, such as the maximum tolerated dose (MTD) and the metronomic or moderate dosage. This may depend on the tumor type, stage, and the mechanism of drug resistance. Curtis, van Berkel, and Frieboes (2018) developed a hybrid model coupling the on‐lattice discrete model of angiogenesis and continuous models for tumor growth, as well as oxygen and nutrients kinetics. This model was used to simulate effective treatment schedules for combinations of chemotherapeutic drugs that reduced tumor size in non‐small cell lung cancer.

Since the number of possible combinations of schedule components (dosage, timing, drug order) can be extremely large, brute‐force computer simulations may be too time‐consuming to perform on a large scale. To overcome this limitation, optimal control techniques have been used in combination with computational models to reduce this computational complexity. Cunningham, Brown, Gatenby, and Stankova (2018) used optimal control together with a continuous model of tumor and T cell populations to obtain optimal schedules of abiraterone delivered to metastatic castrate‐resistant prostate cancer. The dose schedules were selected considering three time‐dependent objective functions (average tumor volume, tumor mass variance, and average T cell population density), which were minimized using a gradient‐based interior‐point optimization algorithm. Fernández and Pola (2019) posted a goal of limiting the systemic drug toxicity while simultaneously minimizing tumor volume. The authors applied an optimal control technique to a continuous model of tumor growth and considered different growth laws, such as Gomperzian growth, the Norton‐Simon hypothesis, and Skipper pharmacodynamics. This study identified an optimal protocol in which the maximum loading infusion rate is applied at the beginning of the treatment protocol, followed by lower infusion rates to maintain the treatment effect. A more detailed analysis of the influence that tumor heterogeneity imposes on optimal chemotherapy protocols was presented by Ledzewicz, Schättler, and Wang (2019). The authors concluded that the upfront dosing of cytotoxic agents at the MTD followed by rest periods is effective if a tumor consists of a homogeneous population of sensitive cells. However, as tumor heterogeneity becomes prevalent and sub‐populations with resistant traits emerge, the protocols with lower‐than‐maximum dose rates can be more effective (adaptive chemotherapy). Nevertheless, if conditions are unfavorable, a resistant cell population may eventually become dominant, and other anticancer therapies (such as immunotherapies) may provide an alternative or be incorporated into combination therapies.

In addition to optimization methods, machine learning methods have also been combined with mathematical models to explore optimal treatment parameters. Sherin, Sohail, and Shujaat (2019) investigated the cytotoxic activity of synthesized gallic acid on ovarian and prostate cancers. The authors used a piecewise recursive Hill model calibrated to experimental data to represent the anticancer effect of the drug and combined it with support vector machine learning algorithms to classify the temporal data and identify optimal drug exposure times that maximize anti‐tumor efficacy. Houy and Grand (2018) integrated the classical continuous pharmacokinetics/pharmacodynamics (PK/PD) model with an artificial intelligence method based on a Monte‐Carlo tree search to optimize the scheduling of temozolomide delivered to brain tumors. The proposed drug scheduling protocols were more efficient than MTD. Li et al. (2013) combined advanced image analysis algorithms with continuous mathematical modeling to determine whether changes in MRI images observed after the first neoadjuvent chemotherapy dose can predict patients' responses to the treatment. MRI signal intensities were quantified to determine a threshold of enhancement between treatment responders and nonresponders. Then, three continuous pharmacokinetic models were used to predict tumor response. Finally, statistical analysis was performed to determine which parameters have prognostic capabilities.

## 4 MODELING TARGETED THERAPY

Targeted therapy is a novel type of anticancer treatment that takes advantage of differences between normal and cancer cells, or of alterations in the tumor stromal microenvironment when compared to a normal tissue (Huang, Shen, Ding, & Geng, 2014; Talwar, Babu, & Raina, 2017). By utilizing molecular abnormalities specific to cancer, targeted therapy is able to more precisely identify and attack cancer cells and is less harmful to normal cells. The hybrid frameworks addressing therapies utilizing the intracellular, vascular and microenvironmental targets are discussed below.

One of the approaches in targeted therapy is to focus on specific intracellular signaling pathways that suppress cell growth or induce cell death. Kim, Kim, Smith, Haura, and Anderson (2018) modeled therapies that target MAPK and PI3K‐AKT signaling in lung cancer. They used hybrid modeling that combines a CA and a set of ODEs to address how tumors respond to these targeted therapies in a heterogeneous microenvironment. The model was calibrated with experimental data and used to predict differences across kinase inhibitor treatments. These simulations suggested rational drug combinations and novel treatment strategies. A different hybrid model was used by Sun et al. (2012) to study the response of brain cancers to tyrosine kinase inhibitors (TKIs). In this model, the CA was used to represent individual tumor cells and EC that form a growing vasculature. Each tumor cell was equipped with an EGFR signaling pathway linked to a cell‐cycle pathway, both described by the ODEs. The microenvironmental factors: glucose, oxygen, fibronectin, VEGF, and transforming growth factor alpha concentrations were modeled using PDEs. This model enabled simulating a dual effect of angiogenesis under TKI treatment: a decrease in tumor invasion due to TKI and an increase in cell survival due to the improved delivery of glucose and oxygen through neo‐vasculature. The interstitial distribution and cellular uptake of the δ‐opioid receptor‐expressing and non‐expressing colon tumors were modeled by Rejniak et al. (2013). The authors integrated a discrete representation of targeted fluorescent agent molecules with a fluid–structure interactions method of regularized Stokeslets. Thus, the model accounted for both the diffusive and advective drug molecule transport. The model simulations showed that tissue penetration by drug molecules of moderate diffusivity are strongly influenced by tissue architecture. The effects of different binding affinities of the targeted therapeutics were also modeled by Vavourakis, Stylianopoulos, and Wijeratne (2018). This model combined a continuous description of a growing tumor, a discrete representation of expanding vasculature, and fluid flow models for vascular, transvascular, and interstitial flows. This hybrid model predicted the relationships between drug binding affinity and the size of the vessel wall pores that may have implications for treatment planning and methods to enhance drug delivery.

Since the tumor‐associated vasculature plays a vital role in tumor growth and progression to metastasis, the drugs that target vasculature are intended to increase tumor suppression. These treatments fall into two categories: the angiogenesis inhibitors (AIs), which bind to angiogenic receptors on the EC and inhibit their proliferation, and the vascular disrupting agents (VDAs), which are designed to selectively target EC and collapse the vascular structure inside the tumor. Both types of vascular‐targeting treatments were modeled by Gevertz (2016) using a combination of CA for individual tumor cells, the Krogh cylinder approach for tumor vasculature, and continuous PDEs solved on a triangular grid for the concentrations of drug molecules and densities of the corresponding receptors. This model was calibrated to pre‐clinical and clinical data and used to explore the hypothesis that AIs and VDAs are complementary treatments, as well as to identify optimal drug dosing strategies. Model simulations suggested a treatment schedule that may have increased clinical anti‐tumor activity compared to currently used treatment protocols. The AIs were also the subject of a study by Glick and Mastroberardino (2017). The authors combined a continuous ODE model with an optimal control approach to determine the optimal drug dosage that can minimize both tumor volume and drug toxicity. Model simulations showed that good tumor control can be achieved using a continuous dosing strategy for anti‐angiogenesis therapy. A novel cell‐based treatment inducing EC apoptosis was modeled by Hendrata and Sudiono (2018). The authors used a CA model to represent individual growing vessels, tumor cells, and mesenchymal stem cells (MSCs) that migrated towards the capillaries and caused their degeneration. The continuous components of the model included the concentration of oxygen, VEGF, and the ECM density. Model simulations showed that the effectiveness of MSC‐mediated anti‐angiogenic therapy was dependent on the dose of applied MSCs and that it took several days to reach the full anti‐endothelium effect.

Recently, a novel class of therapies was developed that can target cells in regions with low levels of oxygen (hypoxia). Since the vasculature in fast‐growing tumors is often chaotic and poorly functional, large subsets of tumor cells can be deprived of nutrients and oxygen. Hypoxic regions can form enclaves in which cells become resistant to chemo‐ and radiotherapy. To overcome the hypoxia barrier, hypoxia‐activated pro‐drugs (HAPs) were designed to be inactive in well‐oxygenated regions but to release cytotoxic agents under low oxygen pressure. A spatially resolved PK/PD model defined on a 3D digitized tissue microregion with explicit vasculature and a continuous description for intracellular and extracellular concentrations of oxygen and drug was developed by Foehrenbacher, Secomb, Wilson, and Hicks (2013). The authors used both normal and tumor digitized tissues to account for the tumor selectivity of cell killing and treatment efficacy. Model simulations suggested that in order to increase drug efficacy, the stability of an active drug and the activation rates of a prodrug should be optimized. The CA model combined with continuous transport equations was used by Hong et al. (2018) to simulate the HAP action in both a 3D multicellular tumor spheroid and a tumor monolayer. Model simulations were used to determine HAP pharmacologic properties that lead to a bystander effect, in which the activated drug is able to locally diffuse and eliminate non‐targeted cells. Such a bystander effect was also investigated by Karolak and Rejniak (2018) using a model that combined discrete nonuniform tumor cells with advection–diffusion–reaction equations representing concentrations of oxygen, inactive HAP, and activated drug. The interstitial fluid flow was modeled using the regularized Stokeslets method. The authors showed that a significant bystander effect is only observable if the diffusion of both the inactive and activated drug is comparable to the diffusion of oxygen. A similar model was used by Wojtkowiak et al. (2015) to investigate the effects of pyruvate injection on changes of tumor oxygenation and HAP activity. The authors concluded that acute pyruvate‐induced increases in tumor hypoxia can be beneficial for improving the clinical efficacy of HAPs.

## 5 MODELING NANOTHERAPY

Nanotherapy is an emerging anticancer treatment strategy in which therapeutic agents are encapsulated in nanoparticles that are then injected into the bloodstream to carry drugs to tumors. Nanoparticles are designed to take advantage of leaky tumor vasculature (due to their size, they do not extravasate in healthy tissues) and to more specifically target tumor cells (due to an engineered carrier ability to bind to specific cell receptors). Thus, nanotherapy can enhance tumor targeting and reduce treatment side effects, the main impediments of classical chemotherapy. Nanoparticles in the circulatory system can adhere to the blood vessels on the tumor site, where they release drugs; alternatively, they can extravasate from the vasculature and decapsulate in the tumor tissue. The released drug or cocktail of drugs can then exert its cytotoxic effect against cancer cells. One way that efficacy of nanodrugs could be improved is by modifying their design, mainly their size and aspect ratio (Gupta, 2015). The hybrid frameworks dealing with optimization of drug structure, intravascular transport, drug release, its interstitial distribution, and tumor cell killing are discussed below.

A mathematical model that evaluates the overall treatment efficacy at various spatial scales was developed by Frieboes, Wu, Lowengrub, Decuzzi, and Cristini (2013). The authors modeled spherical nanoparticles injected into a 2D vascularized tumor and tested particle accumulation at the tumor vessel walls and drug release to the tumor tissue. The tumor vasculature was modeled using an on‐lattice discrete angiogenesis model, while oxygen, nutrients, and drug transport in the tissue were modeled using reaction–diffusion equations. The authors investigated different nanoparticle sizes and showed that large nanoparticles had strong adherence properties but poor spatial distribution, while small nanoparticles were able to reach the tumor core but only at low concentrations. These results indicated that there exists an optimal moderate nanoparticle diameter that leads to balanced nanoparticle accumulation and spatial distribution. This model was then expanded in Curtis, Wu, Lowengrub, Decuzzi, and Frieboes (2015) to study the effect of drug diffusivity on tumor reduction, and in Curtis, Rychahou, Bae, and Frieboes (2016) to investigate the effects of microenvironment acidity on drug release. Moreover, Miller and Frieboes (2019) further extended this model to test how heterogeneity in vascular density influences nanoparticle accumulation and tumor response.

Due to coupling between multiple spatial and temporal scales, the hybrid models of tumor nanotherapy are quite expensive computationally. Therefore, integrating these models with optimization algorithms allows for exploring the entire feasible parameter space in a minimal number of steps. These optimization techniques were implemented by Chamseddine and Kokkolaras (2018) and coupled with a continuous model for nanoparticle accumulation and drug distribution in a 2D tumor structure. The authors used a direct search optimization method, specifically the mesh adaptive direct search (MADS) algorithm (Audet & Dennis, 2006), which allowed them to achieve the optimal nanoparticle design with rigorous convergence properties. The simulated results determined the sizes and aspect ratios of nanoparticles that maximized their accumulation, maximized drug distribution, and enabled quantifying the tradeoff between nanoparticle accumulation and drug distribution through a bi‐objective optimization formulation. Similar optimization methods also work very effectively with hybrid models. Chamseddine, Frieboes, and Kokkolaras (2018) integrated the MADS technique with a hybrid model from Frieboes et al. (2013) to determine the optimal nanoparticle diameters that resulted in maximal intratumoral accumulation of nanoparticles and maximal tumor reduction (Figure 4f). For this computationally expensive model, the authors used a novel surrogate‐assisted optimization technique from Audet, Kokkolaras, Digabel, and Talgorn (2018) to facilitate convergence to the optimal solution.

To account for stochastic treatment parameters such as nanoparticle structure and tumor microenvironment architecture, uncertainty quantification has also been incorporated in modeling of nanoparticle transport. Fronczyk, Guindani, Vannucci, Palange, and Decuzzi (2013) used a Bayesian hierarchical approach integrated with a nanoparticle adhesion model to treat probabilistic distributions of ligand surface density and ligand–receptor bond length. The simulated results showed high robustness in finding optimal nanoparticle properties when high vascular adherence was included. T.‐R. Lee et al. (2014) also considered uncertainties that arise from the tumor microenvironment. To account for inter‐tumor and intra‐patient variations in the vasculature properties, such as vessel diameter and flow velocity, a Bayesian statistical approach was used, leading to a computational framework for designing robust nanotherapeutics.

## 6 MODELING HORMONE THERAPY

Hormone therapy is a form of systemic treatment that targets cells that require hormones to grow. Two prominent examples include breast cancer, which is dependent on estrogens (ER‐positive), and prostate cancer which is dependent on androgens (AR‐positive). Both endocrine therapies targeting the ER pathway in breast cancers and androgen deprivation therapy in prostate cancers are routinely used in the clinic. These therapies have also been subject to mathematical modeling; however, all the models developed so far are either continuous (Chen et al., 2014; Phan, Nguyen, Sharma, & Kuang, 2019) or based on game theory (Zhang, Cunningham, Brown, & Gatenby, 2017) only. This area of research could benefit from utilizing the concept of hybrid modeling frameworks that can address this problem simultaneously at various scales: from designing the administration schedules to the improvement in drug penetration through the tumor tissue, to testing effectiveness in tumor cell receptor binding and internalization.

## 7 MODELING IMMUNOTHERAPY

Immunotherapy is a fast growing field of anticancer treatments that focuses on activating the patient's own immune system. Immunotherapies can be designed to elicit immune response directly in the host tissue or to enhance immune cell functionality ex vivo and then transfer these cells back to the patient body (Grupp & June, 2011). The hybrid modeling frameworks that address different immunotherapy strategies, including the enhancement of cytotoxic T lymphocytes (CTLs), the combination of preventive vaccines and T lymphocytes, and the use of engineered macrophages as drug carriers are discussed below.

One of the main impediments in immunotherapy is the inadequate infiltration of the tumor tissue by immune cells. Even the most potent immune cells cannot exert sufficient anti‐tumor effects if they are not able to reach all tumor cells. Lopez‐Alfonso et al. (2016) combined a CA model and advanced image analysis techniques to simulate immune and epithelial cell interactions in breast lobular epithelium. The authors focused on non‐cancer tissues in order to determine the ranges of normal immune cell infiltrations and better define the physiological states that differentiate between healthy and pathological conditions. The model simulations suggested that the spatial distribution and density of immune cells contain prognostic information that have not been previously considered at diagnosis. Interactions between immune and tumor cells were also modeled by Ozik et al. (2018) by combining the off‐lattice particle model *PhysiCell*, the biotransport model *BioFVM*, and the extreme‐scale model exploration platform *EMEWS*. The authors investigated the effects of different immune cell migration modes and immune‐tumor attachment rates on the size and morphology of the survived tumors. Model simulations showed that the immune response can be significantly improved by decreasing the immune cell migration bias or increasing the attachment lifetime (Figure 4h).

Cancer cells have developed a mechanism to suppress immune cell functionality by expressing the immune checkpoint‐related molecules on their surfaces. One of such molecules is PDL1, which binds to PD‐1 receptors on the activated T cells, resulting in the inhibition of the cytotoxic T cell activity. These deactivated T cells remain inhibited in the tumor microenvironment. Gong et al. (2017) developed a model of tumor–T cells interactions and addressed the pretreatment spatial heterogeneity of PDL1‐positive and ‐negative cells, as well as the role of these spatial patterns in immune checkpoint blockade therapy. This mathematical model combines a 3D CA to represent tumor and immune cells with PDEs to describe cytokine secretion and transport (Figure 4i). The authors proposed a scoring system based on PDL1‐positive and ‐negative cells as a potential biomarker for anti‐PDL1 treatment efficacy. Another therapeutic approach in immunotherapy is to use preventative vaccinations to stimulate the patient's CTLs. P. S. Kim and Lee (2012) developed a model to study the conditions under which CTLs are able to eliminate tumors, such as the size of an initial CTL population, CTL proliferation, and recruitment rates. This model combined the fast‐timescale dynamics of immune interactions in the lymph node described with the delay differential equations and an off‐lattice representation of tumor and T cells at the tumor site. The simulated results showed that a small pool of stimulated CTLs can successfully eradicate a tumor population, implying that a vaccination approach is feasible. Finally, therapeutic strategies have been developed to employ macrophages to deliver drugs to hypovascularized metastatic lesions. Leonard et al. (2016) developed an interdisciplinary approach combining in vitro, in vivo, and in silico methods to assess release and retention dynamics of paclitaxel encapsulated by macrophages. This computational model combined discrete representations of macrophages and tumor vasculature with continuous equations of tumor growth, drug transport, and the transport of various microenvironmental factors such as nutrients and oxygen. The obtained results indicated that the use of macrophages as drug carrier can increase drug efficacy compared to bolus injection and can lead to sustained lesion regression.

## 8 MODELING RADIOTHERAPY

Radiotherapy is a localized treatment in which tumors are exposed to DNA‐damaging ionizing radiation. It is often applied after surgery to prevent tumor recurrence or in combination with other types of therapies, such as chemotherapy, to create synergy in treating aggressive cancers. Several types of radiotherapy are used in the clinic, including external beam radiation therapy, brachytherapy, and intraoperative radiotherapy. However, as with other anticancer therapies, tumor cells may develop resistance. A continuous linear‐quadratic (LQ) model is typically used to calculate beam intensities, but computational studies have been used to complement experiments in developing radiotherapy protocols by selecting effective schedules and the directions of applied radiation beams. The hybrid modeling frameworks that propose radiation protocols utilizing the role of tumor microenvironment, patient‐specific characteristics and strategies to minimize the exposure of healthy organs to damaging radiations are discussed below.

One of the barriers to effective radiotherapy is hypoxia, which is strongly correlated with cell radioresistance. Kempf et al. (2015) studied the effect of cell oxygenation dynamics on tumor radioresistivity by combining a discrete lattice‐free agent‐based model of tumor cells with continuous reaction–diffusion equations of oxygen and nutrient transport (Figure 4d). The authors concluded that optimal radiotherapy protocols should follow cell re‐oxygenation profiles to benefit from oxygen enhancement effects. A different model coupling CA to represent tumor growth with a continuous model of blood and oxygen transport was used by Scott et al. (2016). The authors considered heterogeneity in the properties of tumor vasculature to determine whether vascular architecture can be indicative of radiation response (Figure 4e). In addition to hypoxia, radiosensitivity depends on the cell cycle. López‐Alfonso, Jagiella, Núñez, Herrero, and Drasdo (2014) used an on‐lattice model of two cell populations that differed in their cell‐cycle dynamics, which caused variability in tumor radiosensitivity. The model suggested that heterogenous dosimetries outperform homogenous dosages because of increased radiation effects in the areas occupied by cells with cell‐cycle‐induced radioresistance.

Optimization methods have also been used to plan radiotherapy protocols to maximize efficacy and minimize treatment toxicity. Chan, Mahmoudzadeh, and Perdie (2014) simulated radiation beam intensities to target breast cancers with the objective of minimizing damage to the heart and lungs. Since breathing patterns differ among patients, the authors incorporated the effect of uncertain breathing cycles in the treatment design. The exposure of healthy organs to radiation was modeled based on the patient's breathing cycle and the location of the breast as captured by the CT scans. The result obtained using robust optimization showed enhanced dosimetric performance compared to treatment planning under free breathing. When radiology images are used for modeling, the tumor tissue and neighboring organs are typically discretized into voxels following image resolution, each containing a certain density of cells and vessels. Li, Bissonnette, Purdie, and Chan (2015) considered uncertainty resulting from image voxel values read in PET‐guided radiotherapies and used robust optimization to incorporate the technical errors in the treatment planning process. The authors used intermediate functions to transform a PET scan of the pretreated tumor into the desired dose, and the computed dose was used as an input for the robust optimization model to obtain optimal beamlet intensities. The robust approach showed enhancement in tumor control probability compared to the case when the uncertainty was not considered.

Machine learning methods have also been used to support the personalized design of radiotherapy protocols using population‐based data. Continuous models of tumor growth and radiation response were implemented in Sunassee et al. (2019) and Tariq, Chen, Kirkby, and Jena (2016), and adaptive Bayesian methods were used to infer patient‐specific responses to radiotherapy from the increasing number of data points. These methods allow for the adaptation of radiation protocols during the treatment, taking into account current tumor response.

## 9 MODELING COMBINATION THERAPIES

Since tumors are composed of heterogenous cell populations and are surrounded by a complex and dynamically changing microenvironment, the probability of developing drug resistance is high, which can lead to treatment failure and tumor regrowth or recurrence. Therefore, combining two or more types of therapies that can attack different tumor components or distinct cell subpopulations has been shown to be successful in prolonging patients' survival. However, when multiple treatments are considered, the number of possibilities in designing efficient treatment protocols (the order of therapies, timing between treatments, dosages) can be large. The hybrid modeling frameworks that address combinations of radio‐ and chemotherapy, chemo‐ and immunotherapy, radio‐ and targeted therapies or the use of multiple chemotherapeutic drugs are discussed below.

One of the most commonly modeled treatments is the combination of chemo‐ and radiation therapies. Powathil, Adamson, and Chaplain (2013) and Powathil, Swat, and Chaplain (2015) studied the effects of both the cell‐cycle and oxygen dynamics on radioresistance. The authors integrated a lattice‐based cell model for tumor growth with ODEs describing the cell cycle for each cell, PDEs for oxygen and drug kinetics, and an analytical expression for the radiation effects. Model simulations demonstrated strong coupling between treatment fractionation and the heterogeneity in the cell‐cycle and oxygen distributions, suggesting that these two parameters should be considered when designing optimal treatment schedules. Mao et al. (2018) studied the effects of oxygen dynamics on radiotherapy combined with HAP treatment in colorectal tumors. The authors used a lattice‐based model to represent tumor cells and continuous reaction–diffusion equations to define the transport of drugs, oxygen, and glucose. This 3D model showed a good agreement with experimental tumor growth and treatment response.

In combination therapy, the number of treatment parameters is higher than in monotherapy, and finding effective protocols presents computational challenges. Systematic optimization methods have been used to facilitate the selection of optimized treatment parameters. Gradient‐based approaches were used by Piretto, Delitala, and Ferraro (2018) to find optimal switching between chemotherapy and immunotherapy assuming that the tumor is composed of two cell populations that differ in their resistance capabilities. Gallaher et al. (2014) developed a model for selecting the optimal treatment sequence for metastatic castrate‐resistant prostate cancers. Using a continuous model that couples tumor evolution and changes in bone microenvironment, the authors optimized a treatment protocol based on patient data using genetic algorithm.

Hawkins‐Daarud et al. (2015) modeled the treatment of aggressive glioblastomas that combined resection, radiation, and anti‐angiogenic therapies. The authors used a continuous model with three types of cells that differ in their oxygenation level. Resection was modeled as the removal of a portion of the tumor based on the T1Gd (T1 Weighted Post Contrast) MRI images. The anti‐angiogenic effect was modeled by vessel normalization or the inhibition of angiogenesis, while radiation therapy was defined using the LQ model, with radiosensitivity depending on hypoxia levels. The simulated results provided a testable hypothesis by identifying a subpopulation of patients who could benefit from combining bevacizumab and radiation.

## 10 MODELING ADAPTIVE THERAPY

Typically, cancer treatment protocols are selected based on the tumor stage and grade at the time of diagnosis. The concept of adaptive therapy takes into consideration the dynamic changes in tumor progression while updating protocols during the treatment according to the tumor response. The goal of adaptive therapy can be either to maintain small tumor size, to reduce the emergence of drug resistance, or to control the population of a certain tumor sub‐clone. This is still a young area of research, but continuously growing.

The first mathematical models of adaptive therapy were either exclusively discrete (Zhang et al., 2017; Gallaher, Enriquez‐Navas, Luddy, Gatenby, & Anderson, 2018) or exclusively continuous (West, Ma, & Newton, 2018). However, recent publications incorporate different mathematical methods into hybrid frameworks. Yoon, Vander Velde, Marusyk, and Scott (2018) developed an adaptive therapy model consisting of the continuous description of two cell populations treated by two drugs. Each population was assumed to be sensitive to one drug and resistant to the other. This model was integrated with a gradient‐based selection criterion for optimizing the switching time between the administration of these two drugs with the goal of minimizing cell population. The switching times depend on the cells' proliferation rates and transition rates from sensitive to resistant phases. The optimal switching time was identified to be before one drug becomes ineffective in treating its corresponding cell type. Thus, the study recommended switching to a different drug before tumor regrowth starts.

## 11 CONCLUSIONS

The multiscale models integrating various mathematical and computational approaches are now widely used in modeling tumor development and treatment. The classical hybrid models couple discrete equations to describe individual tumor and stromal cells (agents) and continuous differential equations (ODEs or PDEs) to describe intracellular changes (the cell cycle, protein or receptor levels) or microenvironmental factors (ECM density, concentrations of drugs or oxygen), respectively. Our previous review (Rejniak & Anderson, 2011) provided several examples of such hybrid models of tumor growth. In this updated review, we proposed to extend the definition of hybrid models to include the coupling of either discrete or continuous models with other physics‐based models, such as game theory or fluid dynamics; with data‐driven models, such as machine learning, computer vision or uncertainty quantification; and with optimization models, such as optimal control, optimal design or stochastic optimization. We named this new extended approach the *hybrid modeling frameworks*.

We discussed in this review how the hybrid frameworks are being currently applied to model tumor development and various anticancer therapies, and how the physics‐, data‐ and optimization‐based models can be integrated to bring the added value to solve problems in cancer. However, the future of hybrid frameworks in mathematical oncology will require to build the pipelines for fast integration of patients' data (imaging, genomics, biomarkers) with physics‐based models enhanced by optimization algorithms. One of the future directions indicated by the dotted line in Figure 2, represents applying data‐driven techniques to optimization models. In this approach, the optimization models can provide a library of optimal treatment protocols for various initial conditions (tumor kind, stage, grade, or individual patients' health status) that can be used by machine learning methods to determine the most suitable treatment for a newly diagnosed patient. This is not yet currently possible, since the library of optimal treatment protocols needs to be first validated experimentally (and clinically). In conclusion, the use of hybrid modeling frameworks for predicting optimal treatment protocols brings us a step closer to the development of a quantitative and clinically relevant decision‐making system for personalized medicine.

## CONFLICT OF INTEREST

The authors have declared no conflicts of interest for this article.
